# Supplementary material for: The Systems Biology Research Tool: evolvable open-source software
Source: BMC Syst Biol. 2008 Jun 29;2:55. doi: 10.1186/1752-0509-2-55 (PMC2446383; doi:10.1186/1752-0509-2-55)
Supplement: Additional file 1 — SBRT Archive. An archive of the current version of the Systems Biology Research Tool. [file 1752-0509-2-55-S1.zip › sbrt-1.4.0/doc/developers_guide/api/overview-frame.html]

Overview


All Classes

Packages
  
sbrt
  
sbrt.ext
  
sbrt.ext.cplex
  
sbrt.ext.glpk
  
sbrt.ext.mma
  
sbrt.ext.mma.algebra
  
sbrt.ext.R
  
sbrt.ext.R.stat
  
sbrt.kernel
  
sbrt.kernel.fba
  
sbrt.kernel.fba.analysis
  
sbrt.kernel.fba.comp
  
sbrt.kernel.fba.proc
  
sbrt.kernel.io
  
sbrt.kernel.math
  
sbrt.kernel.math.algebra
  
sbrt.kernel.math.algebra.proc
  
sbrt.kernel.math.comb.comp
  
sbrt.kernel.math.comb.proc
  
sbrt.kernel.math.geom
  
sbrt.kernel.math.geom.comp
  
sbrt.kernel.math.geom.proc
  
sbrt.kernel.math.graph\_theory
  
sbrt.kernel.math.graph\_theory.comp
  
sbrt.kernel.math.graph\_theory.proc
  
sbrt.kernel.math.optimization
  
sbrt.kernel.math.stat
  
sbrt.kernel.math.stat.comp
  
sbrt.kernel.math.stat.proc
  
sbrt.kernel.util
  
sbrt.kernel.utilities.proc
  
sbrt.shell
  
sbrt.shell.gui
  
sbrt.shell.io
  
sbrt.shell.io.fba
  
sbrt.shell.mng
  
sbrt.shell.mng.algebra
  
sbrt.shell.mng.comb
  
sbrt.shell.mng.fba
  
sbrt.shell.mng.geom
  
sbrt.shell.mng.graph\_theory
  
sbrt.shell.mng.stat
  
sbrt.shell.mng.utilities
  
sbrt.shell.proc.fba
  
sbrt.shell.proc.graph\_theory
  
sbrt.shell.text
  
sbrt.shell.text.chem
  
sbrt.shell.text.fba
  
sbrt.shell.text.math
